# Prediction of non-reassuring fetal status and umbilical artery acidosis by the maternal characteristic and ultrasound prior to induction of labor

**DOI:** 10.1186/s12884-021-03972-6

**Published:** 2021-07-06

**Authors:** Jing Lu, Jinna Jiang, Ying Zhou, Qionghua Chen

**Affiliations:** Department of Obstetrics and Gynaecology, Fujian Province, The First Affiliated Hospital of Xiamen University, No.55 Zhenhai Road, Xiamen City, 351000 China

**Keywords:** Middle cerebral artery, Cerebroplacental ratio, Small for gestational age, Non-reassuring fetal status, Fetal acidosis, Ultrasound, Doppler

## Abstract

**Objective:**

To investigate the predictive value of pre-induction digital examination, sonographic measurements and parity for the prediction of non-reassuring fetal status and cord arterial pH < 7.2 prior to the induction of labor (IOL).

**Method:**

This was a prospective observational study, including 384 term pregnancies undergoing IOL. Before the IOL, the Bishop score (BS) by digital examination, sonographic Doppler parameters and the estimated fetal weight (EFW) was assessed. The fetal cord arterial was sampled to measure the pH at delivery. Multivariate logistic regression analysis was performed to identify independent predictors of non-reassuring fetal status and low cord arterial pH.

**Results:**

Forty four cases (11.5%) had non-reassuring fetal status, and 76 cases (19.8%) had fetal cord arterial pH < 7.2. In the non-reassuring fetal status group, the incidence of cord arterial pH < 7.2 was significantly higher than that in the normal fetal heart rate group (χ^2^ = 6.401, *p *= 0.011). Multivariate analysis indicated that significant independent predictors of non-reassuring fetal status were nulliparity (adjusted odds ratio [AOR]: 3.746, *p *= 0.003), EFW < 10^th^ percentile (AOR: 3.764, *p *= 0.003) and cerebroplacental ratio (CPR) < 10^th^ centile (AOR:4.755, *p *< 0.001). In the prediction of non-reassuring fetal status, the performance of the combination of nulliparity and EFW < 10th percentile was improved by the addition of CPR < 10th percentile (AUC: 0.681, (95%CI: 0.636 to 0.742) vs 0.756, (95%CI:0.713 to 0.795)), but the difference was not significant (DeLong test: z = 1.039, *p *= 0.053).. None of the above variables were predictors of cord arterial pH < 7.2.

**Conclusion:**

The risk of fetal acidosis has increased in cases of non-reassuring fetal status. Nulliparity, small for gestational age and CPR < 10th centile are independent predictors for non-reassuring fetal status in term fetuses, though the addition of CPR < 10th centile could not significantly improve the screening accuracy.

## Introduction

Induction of labor (IOL) is a common obstetric management performed in about 20% of all pregnancies with 15% having instrumental delivery and 22% having emergency caesarean section (CS) [1]. As the primary intention of IOL is to achieve safe vaginal delivery, the ability to identify the subclinical compromised fetuses who are prone to the failure of IOL is important for patient counseling, and even more important for the selection of elective CS, avoiding the emergency CS. Therefore, assessment of fetal well-being prior to the IOL may help to detect the fetuses who ultimately require CS or instrumental delivery.

The non-reassuring fetal status may lead to neonatal asphyxia, nervous system injury, cerebral palsy and even perinatal death [2]. The fetal acidosis can cause a decrease in the fetal blood pH value and RCOG's NICE guideline stipulates fetal blood pH < 7.2 as a Class I indication for emergency CS [3]. Although fetal heart rate monitoring for non-reassuring fetal status has been widely used in obstetrics, the incidence of cerebral palsy has not decreased significantly in the past 30 years [4]. The major limitation of fetal heart rate monitoring is the highly subjective interpretation, poor reproducibility [5], and a high false positive rate for the diagnosis of non-reassuring fetal status [6]. Many researches are devoted to the timely and accurate identification of hypoxic fetuses before nervous system injury occurs.

Although cardiotocography (CTG) has been routinely used to assess the fetal well-being prior to the IOL, it represents the last sign of fetal compromise. In contrast, Doppler ultrasonographic assessment of the cerebroplacental ratio (CPR) have been shown to be the early signs of fetal compromise and is associated with adverse outcome [7]. For the small for gestational age (SGA) fetuses, even in the absence of abnormal Doppler, the perinatal outcomes remains worse than the appropriate for gestational age fetuses [8]. At birth, the umbilical artery blood pH value is an objective indicator of the oxygenation status of the fetus. The ability to predict non-reassuring fetal status and fetal acidosis is of great significance to reduce the perinatal complications. Therefore, this research aims to determine whether parity, BS, Doppler parameters and EFW, which can be easily obtained by the ultrasound examination, prior to the IOL could be useful for the prediction of the subsequent non-reassuring fetal status or fetal umbilical artery blood pH value < 7.2.

## Materials and Methods

This was a prospective observational study conducted in a tertiary hospital between January 2020 to December 2020. Pregnant women who were undergoing IOL at term were invited to participate in our study. The inclusion criteria include: (1) singleton pregnancy, (2) gestational age ≥ 37 weeks, (3) cephalic presentation, (4) normal fetal heart rate monitoring on admission. Multiple pregnancies, gestational age < 37 weeks, previous uterine scar and any contraindications to vaginal delivery and labor induction including breech presentation, transverse fetal lie, suspected macrosomia, non-reassuring CTG, placental previa, vasa previa, previous classical hysterotomy and pre-eclampsia were excluded. This study was approved by the Ethics Committee of the First Affiliated Hospital of Xiamen University (XMEC: 2018.153).

Before IOL, the fetal biometrics, including biparietal diameter (BPD), head circumference (HC), abdominal circumference (AC) and femur length (FL), umbilical artery (UA) pulsatility index(PI) and middle cerebral artery (MCA) PI were measured according to the practical guidelines established by the International Society of Ultrasound in Obstetrics and Gynecology [9, 10]. The pulsed wave Doppler of the middle cerebral artery and the umbilical artery were measured in the absence of fetal breathing and movement. The MCA is imaged by the color Doppler at the axial view just below the BPD plane to identify the circle of Willis. Enlarge the image sufficiently, and orientate the probe to make the angle between the ultrasound beam and the direction of blood flow as close to 0° as possible. The sample gate should be placed at the proximal third of the MCA close to the origin from the internal carotid artery, with a sampling volume of 2 mm. The pulsed wave Doppler of the umbilical artery was acquired in the free cord loop. PI was obtained by autotrace measurement, using at least three clear waveforms of the cardiac cycle. CPR was converted into the multiple of median (Mom) of the corresponding gestational week: Median(CPR) =  − 1.3841 + (0.22659 × GA)– (0.003743 × GA^2^), in the formula GA represents the gestational week [11]. The EFW was calculated using the Hadlock Eq.  [12] The percentile of EFW of the corresponding gestational week was calculated using the local reference [13]. In this study, a GE Voluson E8 (GE, Austrian) ultrasound machine equipped with a curvilinear transabdominal probe (C1-5, 2–5 MHz) was used.

IOL was performed followed a standardized departmental protocol. Briefly, intravaginal prostaglandin E_2_ tablet was given to the women with a Bishop score < 6 for cervical ripening before the induction, while the oxytocin and artificial rupture of membranes was used to those with a Bishop score ≥ 6. Fetal heart rate monitoring by CTG was performed during and after IOL. Non-reassuring fetal status is defined as abnormal fetal heart rate monitoring, including repeated fetal heart rate deceleration, fetal tachycardia, bradycardia, and late deceleration [14]. At birth, right before the umbilical cord was cut, the umbilical cord was clamped on the fetal side and the placental side with two forceps. 2 ml umbilical artery blood was drawn using a syringe from the clamped cord, and the pH value was measured immediately using the i-STAT EG7 + cartridge (Abbott, USA). The labor outcome including the mode of delivery, indications of operative delivery was recorded. The results were compared between the non-reassuring fetal status group vs normal fetal heart rate patterns group, and the fetal umbilical artery blood pH < 7.2 vs ≥ 7.2.

Continuous variables were compared unpaired t test or Mann–Whitney U test as appropriate. The prevalence of fetal umbilical artery blood pH < 7.2 between the non-reassuring fetal status group and the normal fetal heart rate patterns group were compared using Chi-square test. Maternal and fetal characteristics were dichotomized for analysis, i.e. parity (nulliparity vs multiparity), EFW and CPR blow the 10^th^ centile or not, the occurrence non-reassuring fetal status or not, the umbilical artery blood pH < 7.2 or not. Univariate analysis was performed to identify the variables associated with non-reassuring fetal status. Multivariate binary logistic regression analysis by forward and backward stepwise conditional elimination method was performed to determine the likelihood of non-reassuring fetal status or umbilical artery blood pH < 7.2. The receiver–operating characteristics (ROC) curve was used to evaluate the accuracy of the prediction model, including the area under the curve, sensitivity and specificity. The area under curve (AUC) was compared using the DeLong test. The statistical analyses were performed using SPSS 20.0 and Medcalc 18.0, with a p value < 0.05 considered statistically significant.

## Results

During the study period, 384 pregnant women who were admitted for IOL were included in the study. The mean maternal age and BMI were 32 years (range: 19 ~ 45 years) and 27.1 kg/m^2^ (range: 19.1 kg/m^2^ ~ 38.6 kg/ m^2^), respectively. 204 women (53.1%) were nulliparous. The primary indications for IOL were postterm pregnancy in 198 (51.6%), gestational diabetes mellitus in 62 (16.1%), suspected macrosomia in 33 (8.6%), fetal growth restriction in 25 (6.5%), oligohydramnios in 24(6.3%), pregnancy-induced hypertension in19(4.9%), history of precipitate labor in 12 (3.1%) and other reasons in 11 (2.9%). 187 women (48.6%) required PGE2 for an unfavorable cervix prior to the IOL.

General characteristics of the subjects are shown in Table 1. Non-reassuring fetal status occurred in 44 cases (11.5%), in which 19 cases had emergency CS, and 25 cases had instrumental delivery. The UA blood pH < 7.2 was seen in 76 cases (19.8%). In the non-reassuring fetal status group, 15 cases (34.1%, 15/44) had UA blood pH < 7.2, whereas in the normal fetal heart rate pattern group, it was seen in 61 cases (17.9%, 61/340). In the group of non-reassuring fetal status, the incidence of umbilical artery blood pH < 7.2 was significantly higher than the normal fetal heart rate pattern group (χ2 = 6.401, *p *= 0.011) (Table 2).

**Table 1:** General characteristics of the subjects

	Normal fetal heart rate pattern	Non-reassuring fetal status	*p*
Number	340	44	
Age	33 (19–45)	32 (23–42)	0.81
BMI (kg/m^2^)	28.0 (19.1–38.6)	26.5 (21.9–38.2)	0.022*
Nulliparity (%)	49.1% (167)	84.1%(37)	< 0.001*
Gestational age	39.6 (37–42)	40.2 (37.1–41.4)	0.031*
Bishop score < 6	46.5% (158)	65.9% (29)	0.015*
UA PI	0.77 ± 0.14	0.81 ± 0.14	0.18
MCA PI	1.38 ± 0.35	1.25 ± 0.31	0.02*
CPR < p10^th^	7.9% (27)	25% (11)	< 0.001*
EFW < p10^th^	7.1% (24)	22.7%(10)	0.001*
Cervical length (cm)	2.4 ± 0.9	2.5 ± 0.9	0.94
Birth weight (g)	3290 ± 430	3104 ± 500	0.007*
Apgar < 7 at 5 min	0	4.5% (2)	0.013*

*BMI* Body mass index, *UA PI* Umbilical artery pulsatility index, *MCA PI* Middle cerebral artery pulsatility index, *CPR* Cerebroplacental ratio, *EFW* Estimated fetal weight

* indicates *p*<0.05.

**Table 2:** The prevalence of umbilical artery blood pH < 7.2 in the group of normal fetal heart rate pattern and non-reassuring fetal status

	Normal fetal heart rate pattern	Non-reassuring fetal status	Total(n)
UA pH < 7.2(n, %)	61(17.9%)*	15(34.1%)*	76
UA pH ≥ 7.2(n, %)	279(82.1%)	29(65.9%)	308
Total(n)	340	44	384

^*^:χ^2^ = 6.401, *p* = 0.011. *UA* Umbilical artery

**Fig. 1:**
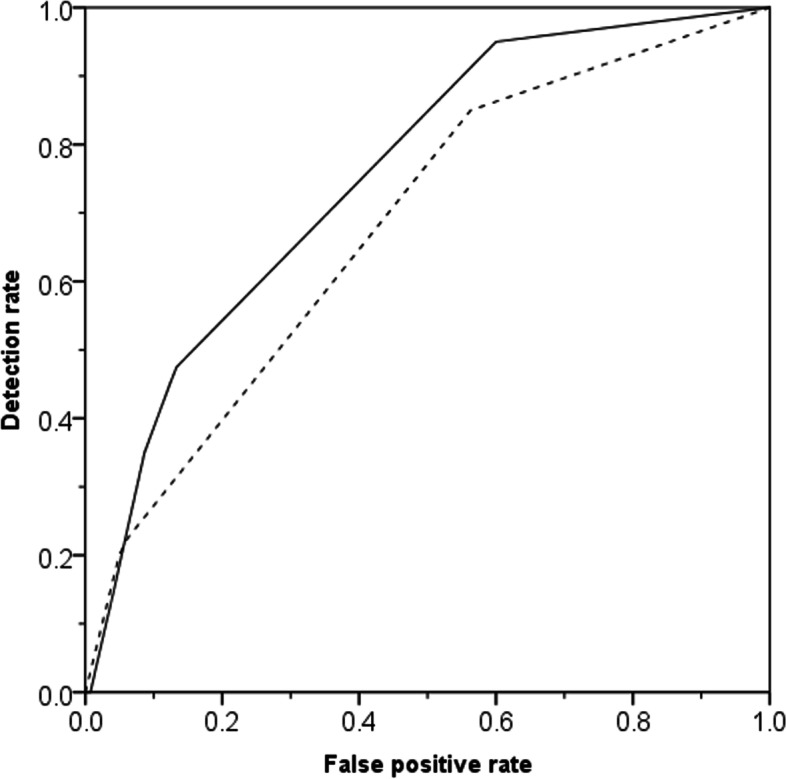
Receiver–operating characteristics analysis for prediction of non-reassuring fetal status by the combination of nulliparity and EFW < 10^th^ centile with (solid line) and without (dashed line) CPR < 10^th^ centile, the area under the curve ROC is 0.756 vs 0.681 (*p *= 0.053)

Univariate analysis indicated that lower BMI (*p *= 0.022), nulliparity (*p *< 0.001), larger gestational age (*p *= 0.031), BS < 6 (*p *= 0.015), lower MCA PI (*p *= 0.02), CPR < 10^th^ centile (*p *< 0.001), EFW < 10^th^ centile (*p *= 0.001), lower birth weight (*p *= 0.007) and Apgar < 7 at 5 min (*p *= 0.013) were associated with non-reassuring fetal status, while UA PI (*p *= 0.18) and cervical length (*p *= 0.94) were not (Table 1). In the multivariate logistic regression analysis, nulliparity (AOR: 3.746, *p *= 0.003), EFW < 10^th^ percentile (AOR:3.764, *p *= 0.003), CPR < 10^th^ percentile (AOR: 4.755, *p *< 0.001) were independent predictors of non-reassuring fetal status, while BS (AOR:1.252, *p *= 0.572) was not (Table 3). In the prediction of the non-reassuring fetal status, the performance of the combination of nulliparity and EFW < 10^th^ percentile was improved by the addition of CPR < 10^th^ percentile (AUC: 0.681, (95%CI: 0.636 to 0.742) vs 0.756, (95%CI:0.713 to 0.795)), but the difference was not significant (DeLong test: z = 1.039, *p *= 0.053). The comparison of the ROC curve is shown in Fig. 1. Combining the three independent predictors, the sensitivity is 95% (95%CI: 83.1% to 99.4%); the specificity is 40% (95%CI: 35.2% to 45%). None of the nulliparity (*p *= 0.955), EFW < 3^rd^ percentile (*p *= 0.88), EFW < 10^th^ percentile (*p *= 0.533), CPR < 1 (*p *= 0.097), CPR < 5^th^ percentile (*p *= 0.866) and MCA PI < 5^th^ percentile (*p *= 0.681) were independent predictors of UA blood pH < 7.2. The pH value of UA blood was not significantly correlated with CPR Mom value (r = 0.074, *p *= 0.145), and was not significantly correlated with the percentile of the EFW (r = 0.004, *p *= 0.94).

**Table 3:** Univariate and multivariate logistic regression analysis for the prediction of non-reassuring fetal status followed IOL

	Univariate analysis			Multivariate analysis		
	OR	95%CI	*p*	AOR	95%CI	*p*
Nulliparity	4.33	1.80–9.92	0.001	3.746	1.572–8.929	0.003
EFW < p10^th^	4.48	1.93–10.44	0.001	3.764	1.555–9.109	0.003
BS < 6	0.46	0.24–0.88	0.015	1.252	0.574–2.732	0.572
CPR < p10^th^	3.81	1.71–8.47	0.001	4.755	2.028–11.148	< 0.001

Formula 1:Log_e_(odds) = -3.651 + 1.321*nulliparity + 1.325 * EFW < 10^th^ centile + 1.559 * CPR < 10^th^ centile

(1 for nulliparity, 0 for multiparity; 1 for EFW < 10^th^ centile, 0 for EFW ≥ 10^th^ centile; 1 for CPR < 10^th^ centile, 0 for CPR ≥ 10^th^ centile)

*AOR* adjusted odds ratio, *EFW* Estimated fetal weight, *BS* Bishop score, *CPR* Cerebroplacental ratio

## Discussion

This study was designed to assess the predictive ability of the maternal characteristics and pre-IOL digital and ultrasound examination for non-reassuring fetal status and decreased UA blood pH. The primary findings are: 1. The incidence of umbilical artery blood pH < 7.2 in the non-reassuring fetal status group was significantly higher than the normal fetal heart rate pattern group; 2. the independent predictors of non-reassuring fetal status included nulliparity, EFW < 10^th^ centile and CPR < 10^th^ centile, while the BS was not; 3. The commonly used Doppler parameters and EFW cannot effectively predict the UA blood pH < 7.2.

Fetal hypoxia and metabolic acidosis can cause permanent neurological damage and even perinatal death. However, it is very challenging to detect fetuses at high risk of intrauterine distress before delivery, because most of them have no obvious complications during pregnancy [15]. The current clinical use of abnormal fetal heart rate monitoring and reversed ductus venosus a-wave is the end-stage of fetal hypoxia [16]. However, abnormal fetal heart rate monitoring cannot sensitively detect the decrease in fetal blood pH. This study found that 80.3% of fetuses with a pH value of cord arterial < 7.2 had no abnormal changes in fetal heart rate. If these fetuses are not delivered in time, the fetal acidemia will be further aggravated, which may eventually lead to fetal distress. This suggests that we need to find more effective predictors of decreased umbilical artery blood pH value. Previous study has shown that besides IOL itself, parity, prior uterine scar, maternal fever and the type of feta heart rate pattern are also significantly predictive of reduced UA pH [17].

Low BS though was associated with non-reassuring fetal status in the univariate analysis, it did not remain so in the multivariate analysis, indicating a confounding factor with nulliparity. Nulliparous women tend to have an unripen cervix and longer interval between the onset of labor and delivery, which increased the risk of non-reassuring fetal status.

The fetal growth restriction is defined as the failure of the fetus to reach its growth potential. The EFW less than the 10^th^ percentile, defined as the small for gestational age (SGA), is regarded as high risk of fetal growth restriction. The SGA term fetuses with the brain sparing effect have a higher risk of abnormal neurological development [18]. Regarding the definition of intrauterine growth restriction, the Delphi consensus defined the EFW less than the 10^th^ percentile with reduced CPR as late-onset fetal growth restriction [19]. CPR Mom value less than the 5^th^ percentile has been used as a proxy of the growth potential [11]. Studies have shown that even for the appropriate for gestational age fetuses with reduced CPR, the fetal blood pH value is significantly decreased [20].

Studies have found that fetuses with oxygen saturation lower than 30% have a significantly reduced MCA PI than those with normal oxygen saturation [21]. In the third trimester, even in the absence of considerable placental failure, mild hypoxia can also occur due to the placental insufficiency. To a lesser extent, UA PI and the fetal weight is normal, but MCA PI and CPR may have been reduced for the subclinical compromised fetuses, [22] for whom the CS rate and the neonatal admission rate are increased [23]. In a large cohort of low-risk term pregnancies, Dall' Asta et al. found reduced CPR in early labor is associated with a higher risk of obstetric intervention due to fetal distress and adverse perinatal outcome [24]. Prior et al. have found that fetuses with CPR less than the 10^th^ percentile have a sixfold increase in the risk of CS due to fetal distress [25]. In the present study, we confined the subjects to the term fetuses, among whom the neurological sequelae caused by premature birth is not a consideration, but the progressively deteriorating metabolic acidosis. The latest RCOG guidelines also recommend that for the term fetuses with brain sparing effects, the baby should be delivered as soon as possible, because it poses the risk of neonatal acidemia [26]. In a large cohort including 1902 term pregnancies, Fiolna et al. has found that a combination of maternal and pregnancy characteristics identified 39% of pregnancies requiring CS for fetal distress at a false positive rate of 10%, while the addition of CPR did not improve the performance of screening [27]. Though fewer variables were assessed in our study, the screening accuracy was similar, with AUC of 0.763 (Fiolna et al.) vs 0.756 (our study); another similarity was the addition of CPR < 10^th^ centile did not significantly improve the screening accuracy. The difference is that EFW < 10^th^ centile was in our model, which may be the reason of a comparable screening accuracy with Fiolna et al.’s.

The strength of this study are the prospective design and the inclusion of a cohort of term fetuses defined according to the local EFW centiles. Regarding the limitations of the study, the Doppler measurement was performed prior to the IOL, while the UA blood pH value was obtained at delivery. There is a time interval between these two measurements, and the fetal acid–base metabolism status may have changed during this period. However, the purpose of this study is to identify subclinical compromised fetuses before IOL, who may not tolerate the uterine contraction and are prone to non-reassuring fetal status [28]. Therefore, the pregnant women could be reasonably counselled and closely monitored before IOL, or even choose a reasonable delivery mode. In addition, in our cohort none of fetuses had cord arterial pH < 7.0, which is an essential condition for causing cerebral palsy [29]. It is not feasible for the analysis of the prediction of severe acidosis. The next question worth thinking is that for the nulliparous women carrying a term SGA fetus, which one is a better indicator for the timing of delivery, reduced CPR or abnormal fetal heart rate. Randomized controlled trials is needed for further investigation.

In conclusion, the risk of fetal acidosis has increased in those have non-reassuring fetal status. Nulliparity, small for gestational age and CPR < 10^th^ centile are independent predictors for non-reassuring fetal status in term fetuses, though the addition of CPR < 10^th^ centile could not significantly improve the screening accuracy.

## Data Availability

The datasets used and/or analysed during the current study are available from the corresponding author on reasonable request.
